# Investigation of the Material Basis Underlying the Correlation between Presbycusis and Kidney Deficiency in Traditional Chinese Medicine via GC/MS Metabolomics

**DOI:** 10.1155/2013/762092

**Published:** 2013-11-25

**Authors:** Yang Dong, Yue Ding, Pu-Zhao Liu, Hai-Yan Song, Yu-Ping Zhao, Ming Li, Jian-Rong Shi

**Affiliations:** ^1^Experimental Teaching Center, Shanghai University of Traditional Chinese Medicine, Shanghai 201203, China; ^2^Department of Otolaryngology-Head & Neck Surgery, Shanghai Yueyang Hospital, Shanghai 200437, China; ^3^Basic Medical College, Shanghai University of Traditional Chinese Medicine, Shanghai 201203, China

## Abstract

*Objective*. To investigate the correlation between presbycusis and kidney deficiency as defined by traditional Chinese medicine (TCM) and its material basis from the perspective of metabolism. *Methods*. Pure-tone audiometry was used to test auditory function. A kidney deficiency symptom scoring table was used to measure the kidney deficiency accumulated scores of the research subjects. Gas chromatography/mass spectrometry (GC/MS) was used to measure the metabolites in the urine samples from 11 presbycusis patients and 9 elderly people with normal hearing. *Results*. Hearing loss in the elderly was positively correlated with kidney deficiency score in TCM. There were significant differences in urine metabolite profile between the presbycusis patients and the controls. A total of 23 differentially expressed metabolites were found. Kyoto Encyclopedia of Genes and Genomes (KEGG) pathway analysis showed that these metabolites were related to glutathione metabolism, amino acid metabolism, glucose metabolism, the *N*-methyl-D-aspartic acid (NMDA) receptor pathway, and the **γ**-aminobutyric acid (GABA) receptor pathway. *Conclusion*. Glutathione metabolism, amino acid metabolism, glucose metabolism, NMDA receptors, and GABA receptors may be related to the pathogenesis of presbycusis and may be the material basis underlying the correlation between presbycusis and kidney deficiency in TCM.

## 1. Introduction

Age-related hearing loss, or presbycusis, is the emergence of binaural, symmetric, and slowly progressing sensorineural hearing loss due to auditory organ aging and degeneration and is a complex disease caused by various factors. The theory of traditional Chinese medicine (TCM) states that the aging of the body is the physiological and pathological outcome of the phenomena that a person experiences throughout life, causing the essence and Qi of the kidneys to dissipate continuously, the yin and yang to become imbalanced, and the functions of the internal organs to gradually decline. This theory can be called a kidney deficiency-based model of aging. “Su Wen • Chapter on the discussion of ancient nature” says, “Males…at 40 years old show weakened kidney energy, falling hair, and withering teeth; …at 56 years old…they show kidney deficiency and aged body appearance.” “Elementary Medicine,” written in the Ming Dynasty, says, “Once people reach middle age, kidney energy starts to decline.” Both stress the correlation between aging and kidney deficiency. Professor Ziyin Shen has found that aging and kidney deficiency both involve significant changes in many indicators of the nervous-endocrine-immune networks, especially of the hypothalamus-pituitary-adrenal cortex-thymus axis. He thus proposed the theory that “the essence of aging is physiological kidney deficiency.” By testing the effects of various drugs, he also discovered that a kidney deficiency rat model (aging rats) shows abnormalities in the nervous-endocrine-immune and nervous-endocrine-bone metabolism pathways and that nourishing the kidneys could correct the decreased functions of these networks [1].

Presbycusis is a degenerative disease due to the aging of the auditory system and is part of the manifestations of the aging body. Therefore, presbycusis can also be seen as a model of hearing loss due to kidney deficiency. Epidemiological surveys show that using >25 dB as the standard, the prevalence of presbycusis is 28.7% in males 60–69 years old and 17% in females 60–69 years old and that the incidence rate doubles with each increase of 10 years [2]. The kidney deficiency indicators of the elderly have similar patterns of change. The results of a survey of 2137 people in the Changning District of Shanghai who were 60 years of age or older showed that the prevalence of kidney deficiency was 78.8% and the incidence increased by 6%–10% for each increase of 5 years [3]. These data suggest that kidney deficiency is closely related to the occurrence of presbycusis.

Metabolomics is an emerging genomic discipline following in the path of genomics, transcriptomics, and proteomics. Metabolomics uses high-throughput and highly sensitive analytical techniques in combination with chemometric methods to analyze metabolites in body fluids. Metabolomics can integrate all information about metabolites and compare metabolic properties in order to identify early sensitive biomarkers. Metabolomics can help in the analysis of pathological processes and in clinical diagnosis. Metabolomic technology has been used for the research of Alzheimer's disease (AD), asthma, cancer, and many other diseases and is expected to become an important means of screening and diagnosis of clinical diseases. ^1^H-nuclear magnetic resonance- (NMR-) based metabolite profiling of serum may be useful for the effective diagnosis of asthma and an improved understanding of its pathogenesis. Multivariate statistical analysis has shown a clear metabolite distinction between patients with asthma and healthy subjects. Serum of asthma patients is characterized by increased methionine, glutamine, and histidine and by decreased formate, methanol, acetate, choline, *O*-phosphocholine, arginine, and glucose, which are involved in hypermethylation, response to hypoxia, and immune response [4]. Czech et al. [5] analyzed CSF samples from 79 AD patients and 51 healthy controls by gas and liquid chromatography-tandem mass spectrometry (GC-MS and LC-MS/MS), which identified 343 different analytes. Their results showed that increased cortisol seemed to be related to the progression of AD. Increased cysteine associated with decreased uridine was the best combination to identify light AD, with a specificity and sensitivity above 75%. In addition, quantitative analysis of tumor growth and metabolomic data by NMR-metabolomics showed that the combination of everolimus and irinotecan was more beneficial in BRAF/PIK3CA-mutant HT29 tumor xenografts, where it had an additive effect, than in KRAS/PIK3CA-mutant HCT116 tumor xenografts, where it had a less additive effect [6].

The high-throughput feature of metabolomics coincides with the overall concept of TCM. Metabolomics has begun to be used in TCM research, including studies on the basic theory of TCM, TCM syndrome research, and research on traditional Chinese medications, and has become an important research method in the study of TCM. Cui et al. [7] explored the urine biochemistry features of syndromes of TCM, such as the syndrome of stagnation of liver Qi, spleen deficiency, liver Qi stagnation, and spleen deficiency (LSSDS) in suboptimal health status (SHS) individuals using ^1^H-NMR. They concluded that there were differences in the ^1^H-NMR-metabolic spectrum of the urine samples of the four syndrome groups, and the specific metabolic products of LSSDS in SHS individuals were identified from metabonomic analysis. Wu et al. [8] investigated the metabolic profile of urinary carbohydrates using GC/MS and multivariate statistical analysis. The combination of GC/MS and K-orthogonal partial least squares (OPLS)/subwindow permutation analysis allowed them to characterize the urinary carbohydrate metabolic characterization of diabetes patients with different TCM syndromes, including biomarkers different from nondiabetic patients. The method presented by Wu et al. is thought to be a complement or an alternative to TCM syndrome research. However, metabolomics technologies have not yet been used in the study of the TCM visceral-state doctrine.

 In this study, GC/MS was used to compare the metabolite profile of urine samples from presbycusis patients and people with normal hearing and to screen differentially expressed metabolites. By performing preliminary analysis of the biological significance of these differences and the correlation analysis between kidney deficiency defined by TCM and presbycusis, we investigated the material basis of this correlation from a metabolic perspective and provide new experimental evidence for modern-biological research into the TCM theory that the kidneys control the ears.

## 2. Materials and Methods

### 2.1. Subjects

 From November 2009 to July 2010, 11 presbycusis patients were recruited at Otolaryngological Department and 9 elderly people with normal hearing were recruited at Center for Physical Examination in Shanghai Yueyang Hospital of Integrated Traditional Chinese and Western Medicine. The subjects were 60 to 84 years old. In an acoustic room (ambient noise less than 20 dB), pure-tone audiometry was conducted with an AC40 audiometer (Denmark). Patients with the better ear showing an average hearing threshold >25 dB HL at 0.5, 1, 2, and 4 kHz were diagnosed with deafness. An AZ26 acoustic impedance audiometer (Denmark) was used for acoustic impedance side listening examination to test middle ear function, in order to exclude middle ear diseases.

### 2.2. Diagnostic Criteria

 According to the criteria listed in “Practice of Otorhinolaryngology” by Xuanzhao Huang, after the exclusion of other causes, elderly people ≥60 years of age with binaural progressive sensorineural hearing loss can be diagnosed as presbycusis; in pure-tone audiometry, an average hearing threshold >25 dB HL at 0.5, 1, 2, and 4 kHz indicates deafness.

### 2.3. Inclusion and Exclusion Criteria

#### 2.3.1. Inclusion Criteria

Patients who were between 60 and 84 years and fulfilled the diagnostic criteria for presbycusis were included.

#### 2.3.2. Exclusion Criteria

Patients who were younger than 60 or older than 84 years; with drug-induced hearing loss; noise-induced hearing loss; autoimmune hearing loss; congenital deafness; hearing loss due to outer or middle ear diseases; deafness caused by hypothyroidism; and severe primary diseases in the liver, kidney, endocrine, hematopoietic, or other systems or mental illness were excluded.

### 2.4. Scoring of the Kidney Deficiency Symptoms

A scoring table (Table 1) for kidney deficiency symptoms was generated according to “Standards of practice for evaluating the grades of kidney deficiency syndrome differentiation factors” [9] by Professor Shilin Yan and “Standards of reference of TCM deficiency syndrome” revised by the Chinese Integrated Traditional and Western Medicine Deficiency Syndrome and Geriatrics Research Professional Committee. These symptoms included soreness and pain in the waist and knee (except traumatic injuries), soreness in the shin, weak knee or heel pain, tinnitus or deafness, hair loss or loose teeth, forgetfulness, postvoiding dribble, or urinary incontinence, sexual dysfunction, pale tongue, and thin pulse. A score of 0 indicated asymptomatic conditions, 2 represented mild or occasional symptoms, and 4 indicated severe or frequent symptoms. Tongue and pulse manifestations characteristic of kidney deficiency each added 2 points. The scoring table was approved by both TCM diagnosis experts and otolaryngology clinical specialists. Clinicians in the Department of Otorhinolaryngology at Yueyang Hospital questioned and examined the patients and filled out the scoring table.

### 2.5. Urine Sample Collection and Processing

 Urine samples of patients were collected at 8 a.m. to 9 a.m. The patients were asked to be empty stomach and without medicine intervention before urine collection. Urine samples were taken 2 h before the experiment and were thawed at room temperature with shaking. One milliliter of urine sample was placed in a centrifuge tube and centrifuged at 12,000 rpm for 10 min. Then, 200 *μ*L of the supernatant was removed and placed in a 1.5 mL centrifuge tube, followed by 30 sec of oscillation. The supernatant was then left at 37°C and reacted for 15 min to remove urea. Methanol and 13-methyl myristate were added, followed by 1 min of oscillation. After centrifugation at 13,000 rpm for 10 min, 200 *μ*L of the supernatant was removed, placed in a GC vial, and dried in a stream of nitrogen at room temperature. Methoxamine was then added, and the vial was capped, followed by 1 min of oscillation. The vial was shaken at 200 rpm at 30°C for 90 min for the methoxamine carbonyl blocking reaction. Subsequently, the vial was opened to add *N,O*-bis(trimethylsilyl)trifluoroacetamide + 1% trimethylchlorosilane, sealed, and oscillated for 30 sec. Then the vial was opened again, *n*-butane was added, and it was sealed, and oscillated for 30 sec.

### 2.6. GC/MS Analysis and Condition Setup

The methods of GC/MS were similar to previous report [10]. 1 *μ*L of the trimethylsilyl-derived urine sample extracts was used for GC/MS analysis. The GC conditions were as follows: inlet temperature 260°C, injection volume 1.0 *μ*L, splitless injection, high-purity helium carrier gas (99.999%), flow rate 1.0 mL/min, interface temperature 280°C, and a chromatographic column (DB-5MS capillary column) temperature elevation program as shown in Table 2. The MS conditions were as follows: ion source temperature 230°C, quadrupole temperature 150°C, solvent delay 5 min, electron impact ionization voltage 70 eV, MS full scan range 30–550 *m*/*z*, and full scan mode.

### 2.7. Data Processing and Pattern Recognition

#### 2.7.1. Data Preprocessing

Raw data were converted into NetCDF format by the software carried by the Agilent MSD workstation and then imported into R software and processed using the XCMS Toolbox (http://metlin.scripps.edu/xcms/). After the calculation processes, such as baseline correction, peak identification, and peak alignment, a three-dimensional matrix was finally obtained, consisting of the specified peak index (pairs of retention time and mass/charge ratio), sample name, and peak area, and introduced into the SIMCA-P11.5 software program for multidimensional statistical analysis.

#### 2.7.2. Pattern Recognition

The resulting three-dimensional matrix was introduced into SIMCA-P11.5, and principal component analysis (PCA) was conducted after centralization and normalization in order to examine the overall metabolic profile among each group of samples and to observe the sample aggregation, discretization, and outliers. Subsequently, partial least square-discriminant analysis (PLS-DA) was used to identify the main differential variables causing this type of aggregation and discretization. The PLS-DA model went through seven cycles of mutual authentication with the 1/7 sample exclusion method. To identify and distinguish the metabolites making large contributions to the metabolite profile of each group, an OPLS-discriminant analysis (DA) model was used to find the most relevant differentially expressed metabolites for presbycusis, and the threshold of VIP greater than 1.0 and P greater than 0.5 indicated differentially expressed metabolites that were significant in multidimensional statistics.

#### 2.7.3. One-Dimensional Statistical Analysis

One-dimensional verification using the average rank and fold change and Mann-Whitney *U* test was used to validate the multidimensional statistical results. Metabolites whose VIP was >1.0 and whose *P* value was <0.05 in one-dimensional statistical analysis were the final differentially expressed metabolites.

#### 2.7.4. Identification of Differentially Expressed Metabolites

The variables were identified by National Institute of Standards and Technology (NIST) database using the AMD IS_32 and MS Search v.1.7 programs. After identifying the differentially expressed variables (mass/charge ratio, retention time) in the spectrum, the corresponding metabolites were retrieved from the spectral database, allowing us to identify and distinguish the differentially expressed metabolites.

## 3. Results

### 3.1. Kidney Deficiency Accumulated Points

**Figure 1:**
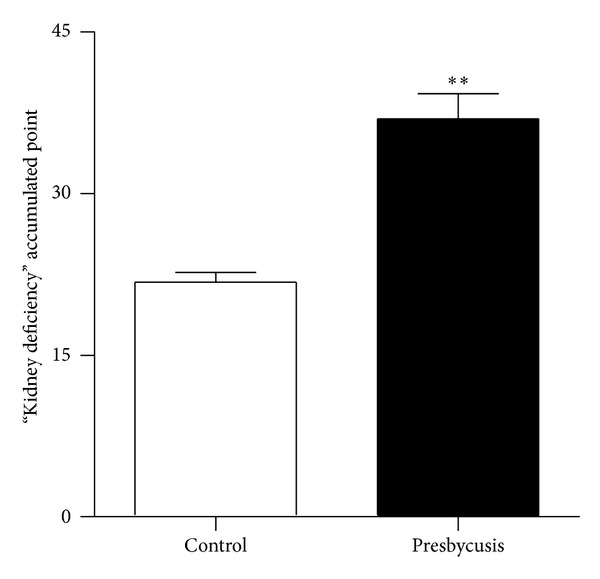
Kidney deficiency accumulated points. **P < 0.01, as compared to control group.

The general clinical data is shown in Table 3. The average age of control group and presbycusis group had no significant difference.  Figure 1 shows that the kidney deficiency accumulated points of the presbycusis group were significantly higher than those of the control group of elderly people without deafness (*P* < 0.01). In addition, Pearson correlation analysis showed that kidney deficiency was positively correlated with hearing loss in patients with presbycusis (*r* = 0.766, *P* < 0.05).

### 3.2. Total Ion Current Chromatogram of Urine Analysis

**Figure 2:**
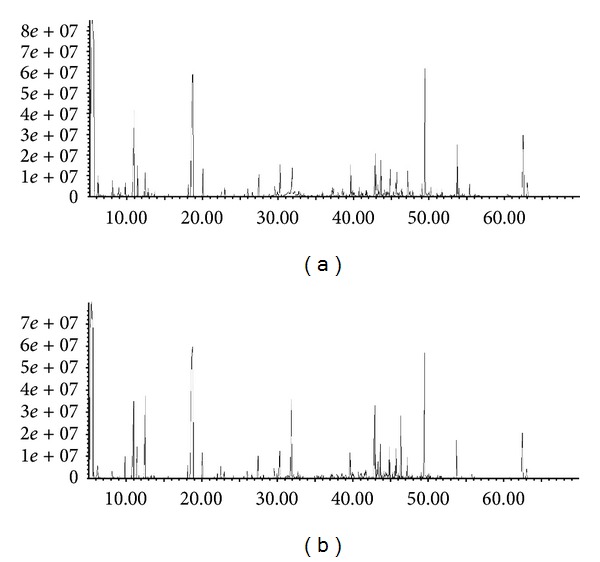
Ion current chromatograms of urine samples from the control group of elderly people without hearing loss (a) and presbycusis patients (b).

The total ion current chromatograms of the urine samples from elderly people with normal hearing and elderly patients with presbycusis were obtained after GC/MS analysis, as shown in Figure 2.

### 3.3. OPLS-DA

**Figure 3:**
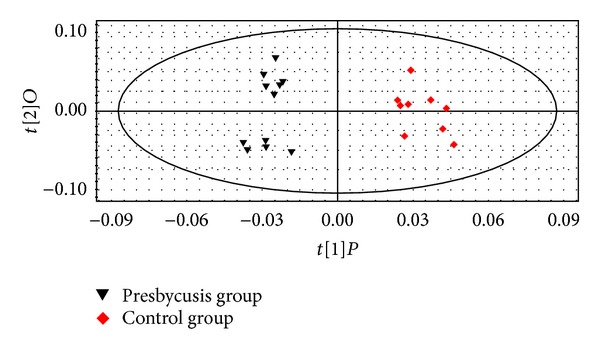
OPLS-DA chart of the urine metabolomes of presbycusis patients and the control group of elderly people without hearing loss.

**Figure 4:**
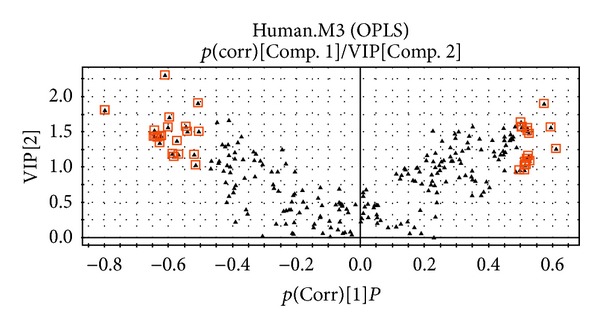
V-PLOT chart of the urine metabolomes of presbycusis patients and the control group of elderly people without hearing loss.

 OPLS-DA was used to identify metabolites that made large contributions to the metabolic profile of each group. The samples of elderly patients with presbycusis could be completely separated from samples of elderly people with normal hearing, yielding a good separation model (Figure 3). In Figure 4, the substances in the red box are considered the differentially expressed metabolites most relevant to presbycusis. 

### 3.4. Differentially Expressed Metabolites

**Figure 5:**
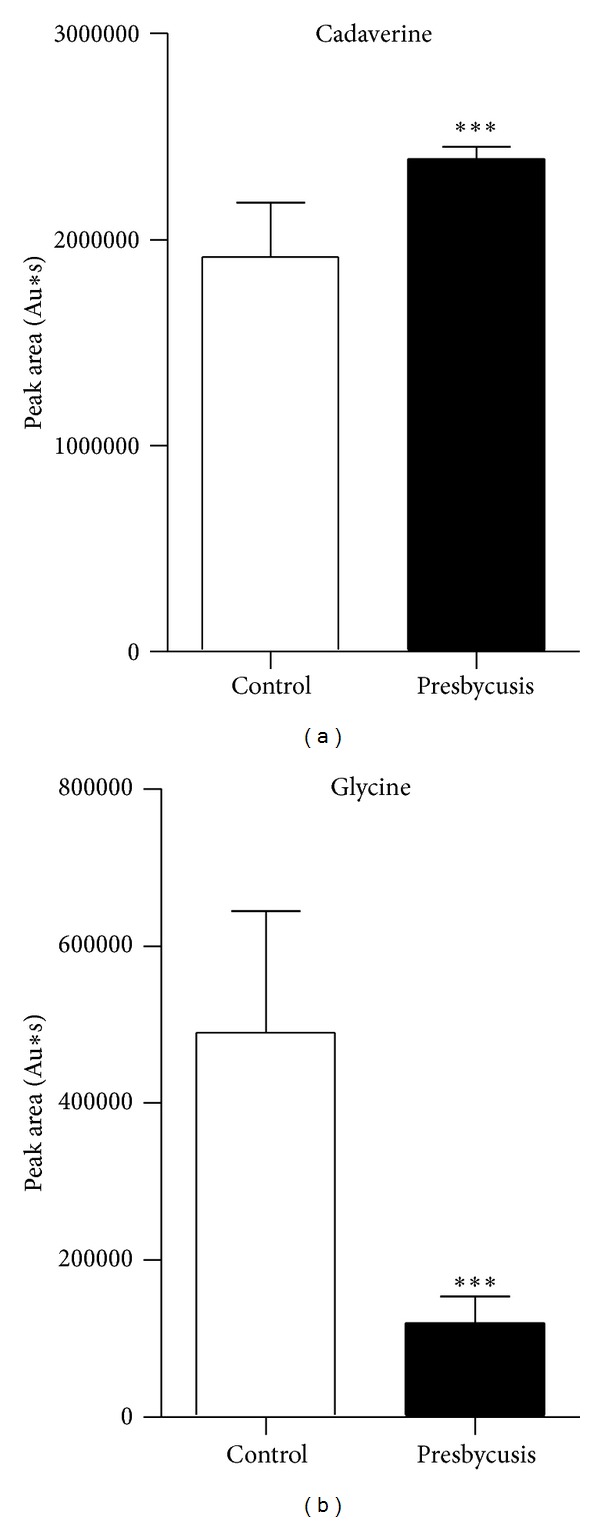
Peak area of cadaverine (a) and glycine (b). ***P < 0.001, as compared to the control group.

The NIST Mass Spectral Database was used to identify all common endogenous metabolites. A total of 23 differentially expressed metabolites were identified: trifluoromethyl-bis-(trimethylsilyl)methyl ketone, butyric acid, 3-ethyl-6-pentamethyldisilyloxyoctane, piperazine, propanedioic acid, ethylenediamine, (R*,S*)-3,4-dihydroxybutanoic acid, 1,4-butanediamine, propionic acid, dodecanoic acid, cadaverine, 1,3,2-dioxaborolane, benzeneacetic acid, d-xylose, pentaric acid, 3-(3-hydroxyphenyl)-3-hydroxypropionic acid, d-mannitol, glucose oxime hexakis, d-gluconic acid, glycine, lyxose, ^1^H-indole-3-acetic acid, and allonic acid. To gain some insight into the functions of these substances, the Kyoto Encyclopedia of Genes and Genomes (KEGG) database was used to investigate the metabolic pathways in which they are involved. The results showed that these substances were related to glutathione metabolism, amino acid metabolism, glucose metabolism, *N*-methyl-d-aspartic acid (NMDA) receptor, and *γ*-aminobutyric acid (GABA) receptor (Table 4). As shown in Figure 5, the results of Cadaverine and Glycine had significant difference between presbycusis group and control group (*P* < 0.001).

## 4. Discussion

The theory about the “kidneys controlling ears” is an important component of the TCM visceral-state doctrine. During the “Huang Di Nei Jing,” it was written that “the ear is the aperture of the kidney” and “kidneys control the ears, their apertures are the ears.” From the perspective of their physiological relationships, “the kidney Qi passes to the ear, and harmony in the kidney enables the hearing of five notes of the pentatonic scale” (“Divine Pivot Measurement of the pulse length”). From the pathological perspective, “Sufficient kidney Qi enables hearing and sensitizes hearing. Under the condition of fatigue, the essence and Qi first become deficient, the Qi of the four seasons can enter from the outside, and the seven emotional states are damaged in the inside. This situation leads to hearing loss, deafness, and tinnitus" (“Zhengzhi Huibu” volume four). Thus, the relationship between the kidney and hearing loss is one of the core tenets of TCM.

 Presbycusis is the manifestation of aging in hearing function. TCM theory suggests that the essence of aging is kidney deficiency. The kidney opens into the ear, and medicine nourishing the kidney can delay age-related changes in certain functions of the body by regulating all levels of neurotransmitters or aging-related hormones in the pituitary, thus preventing presbycusis [11]. Presbycusis is undoubtedly the perfect model to reflect the relationship between the kidney and ear in TCM. In this study, the result showed that the presbycusis group had significantly more kidney deficiency accumulated points than elderly with normal hearing. The correlation analysis showed that presbycusis and kidney deficiency (according to TCM criteria) were correlated. Therefore, the investigation of the material basis of this correlation is necessary to discover the modern-biological mechanisms of presbycusis and test the scientific merit of the TCM kidney-control-ear theory.

**Figure 6:**
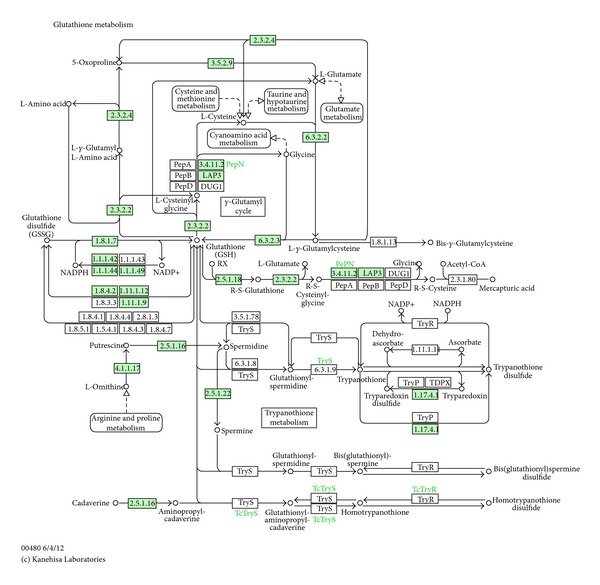
Glutathione metabolism pathway diagram (Human) by KEGG.

 This study used GC/MS to perform a metabolomic analysis of urine samples from presbycusis patients and identified 23 differentially expressed metabolites. KEGG pathway analysis revealed that these metabolites were mainly related to the glutathione metabolism pathway, amino acid metabolism, and glucose metabolism. The results show that the level of glycine, a metabolite related to glutathione metabolism, was decreased and the levels of 3-ethyl-6-pentamethyldisilyloxyoctane, cadaverine, and 1,4-butanediamine were increased in presbycusis patients. Cadaverine is a decarboxylation product of lysine that can regulate tryptophan and its receptor, thereby participating in glutathione metabolism. Glycine, glutamic acid, and cysteine form the short peptide glutathione, which is present in almost every cell of the body and is an important part of the body's antioxidant system. Glutathione scavenges free radicals and protects cells from damage caused by free radicals. Glutathione deficiency is a major cause of oxidative stress during the aging process, and increasing the uptake of the glutathione precursors glycine and cysteine can restore glutathione synthesis and concentration, reducing oxidative stress and oxidative damage during aging [12]. The glutathione metabolism pathway (including glycine and cadaverine) diagram is shown in Figure 6. Glutathione and its related enzymes are related to hearing loss associated with aging. The glutathione levels in the auditory nerves of aging 24-month-old F344 rats are significantly lower than those of 3-month-old rats [13]. In aging F344 rats, the expression levels of multiple genes related to glutathione, such as glutathione peroxidase 3, glutathione peroxidase 6, glutathione S-transferase (GST) kappa-1, and glutathione reductase, are upregulated compared with young rats, suggesting that the glutathione antioxidant system is the first barrier against oxidative damage to the cochlea and mitigating presbycusis [14]. Glutathione-binding protein is a marker of oxidative stress, and its expression in the mouse cochlea increases with age [15]. In addition, the enzymes involved in glutathione metabolism, including glutathione peroxidase, glutathione reductase, and particularly GST, have important roles in the antioxidant protection of the cochlea. Bared et al. [16] found that people with the GST *μ*1 (*GSTM1*) null genotype cannot bind specific metabolites, and their high-frequency distortion-product otoacoustic emission amplitude is lower than that of people who express the *GSTM1* gene, suggesting that the *GSTM1* null genotype leads to a predisposition to presbycusis. The glutathione metabolism pathway diagram was shown in Figure 6.

 The differentially expressed metabolite 3-ethyl-6-pentamethyldisilyloxyoctane is related to GABAergic synapses, and 1,4-butanediamine can act on the NMDA receptor. The impairment of inhibitory GABAergic neurotransmission in the inferior colliculus may contribute to the abnormal auditory perception and processing seen in neural presbycusis [17]. There is strong evidence of an age-related change in GABA(A) receptor composition, which may reflect a compensatory upregulation of inhibitory function in the face of significant loss of presynaptic GABA release [18]. Osumi et al. [19], using the combination of microarray, qPCR, and in situ hybridization, showed that the decline of GluN1 in the inferior colliculus of aging animals might have a key role in the pathogenesis of presbycusis. They showed that from the perspective of metabolism, the NMDA receptor and GABA receptor may be involved in the occurrence of presbycusis. In the nervous system, glutathione can act as a neurotransmitter or modulator of the glutamate receptor and the NMDA receptor, and it can also act directly on the GABA receptor and the glycine receptor [20, 21]. Thus, it is possible that glutathione metabolism changes during the course of presbycusis, subsequently affecting the concentrations of the metabolites 1,4-butanediamine and 3-ethyl-6-pentamethyldisilyloxyoctane through the NMDA receptor and GABA receptor. 

 Our study showed that various metabolites related to amino acid metabolism were significantly different between the presbycusis group and the normal control group. The amino acid levels in the body are related to the body's aging. Abnormalities in the plasma supply of phenylalanine and tyrosine are related to the occurrence and deterioration of cognitive dysfunction in the elderly [22]. In addition, glutathione can participate in the transportation of amino acids *in vivo*, as well as glucose metabolism and the Krebs cycle. Some differentially expressed metabolites were also found to be related to these metabolic pathways. These metabolic pathways may interact to form interrelated metabolic regulatory networks.

## 5. Conclusions

In summary, by OPLS-DA analysis, we detected significant differences in the urine metabolic profiles between patients with presbycusis and elderly people with normal hearing. A total of 23 differentially expressed metabolites were identified. Preliminary analysis showed that these differentially expressed metabolites are related to glutathione metabolism, amino acid metabolism, glucose metabolism, NMDA receptor activity, and GABA receptor activity. These metabolic pathways may be the material basis for the correlation between presbycusis and kidney deficiency as posited in TCM. This study provides a new basis for the modern-biological mechanical investigation of the TCM kidney/ear theory from the perspective of metabolism. Metabolomic detection based on GC/MS could become a valuable tool for the diagnosis of presbycusis and for testing the TCM kidney/ear theory of the visceral-state doctrine.

## Figures and Tables

**Table 1:** Clinical questionnaire about kidney deficiency score.

Number	Symptoms	Evaluation
1	Hearing loss	○ None ○ Yes
2	Tinnitus	○ None ○ Occasional ○ Frequent
3	Soreness and pain in the waist and knee	○ None ○ Occasional ○ Frequent
4	Dysphoria and wakefulness	○ None ○ Occasional ○ Frequent
5	Forgetfulness	○ None ○ Occasional ○ Frequent
6	Dizziness	○ None ○ Occasional ○ Frequent
7	Failing eyesight	○ None ○ Occasional ○ Frequent
8	Hair loss	○ None ○ Mild ○ Severe
9	White hair	○ None ○ Some ○ Total
20	Luxated tooth	○ None ○ Mild ○ Severe
21	Enuresis nocturna	○ None ○ Occasional ○ Frequent
22	Postvoiding dribble or urinary incontinence	○ None ○ Occasional ○ Frequent
23	Frequent micturition in the day	○ None ○ Occasional ○ Frequent
24	Sexual dysfunction	○ None ○ Mild ○ Severe
25	Mental fatigue	○ None ○ Occasional ○ Frequent
26	Extreme chilliness	○ None ○ Occasional ○ Frequent
27	Lassitude	○ None ○ Occasional ○ Frequent
28	Thirsty	○ None ○ Occasional ○ Frequent
29	Five frustrating heat	○ None ○ Occasional ○ Frequent
30	Night sweat	○ None ○ Occasional ○ Frequent
33	Facial complexion	○ Pale white ○ Dim ○ Bright white ○ Dark
34	Tongue nature	○ Pale ○ Enlarged
35	Coating on the tongue	○ Thin and white ○ Less fur ○ White and greasy
36	Pulse condition	○ Thready and weak ○ Thready and rapid ○ Deep and thready ○ CHI-pulse being weak

Methods of score: “none” is zero point, “mild, occasional, yes, or some” is 2 points, “total, frequent, or severe” is 4 points. Each item in facial complexion, tongue nature, coating on the tongue, and pulse condition is 2 points.

**Table 2:** GC/MS chromatography column oven temperature elevation program.

Rate (°C/min)	Temperature (°C)	Hold time (min)
	70	2
2.5	160	0
5	240	16

**Table 3:** Clinical data of patients (Mean ± SEM).

Group	*N*	Gender	Age (year)	Hearing threshold (dB)
Control group	9	Male 5 Female 4	65.0 ± 1.8	19.7 ± 1.3
Presbycusis group	11	Male 6 Female 5	66.5 ± 2.4	44.7 ± 4.2**

**P < 0.01, as compared to the control group.

**Table 4:** Differentially expressed substances and the related pathways.

Differentially expressed substance	Fold change	*P* values	Related pathway
1,4-Butanediamine	+1.79	0.0031	NMDA receptor
3-Ethyl-6-pentamethyldisilyloxyoctane	+1.67	0.0056	GABA receptor
d-Xylose	+1.67	0.0056	Glucose metabolism
1,3,2-Dioxaborolane	+1.64	0.0067	Unknown
Cadaverine	+1.60	0.025	Glutathione metabolism
Butyric acid	+1.57	0.0095	Amino acid metabolism
Piperazine	+1.54	0.0112	Amino acid metabolism (histamine H1 receptor)
Trifluoromethyl-bis-(trimethylsilyl)methyl ketone	+1.50	0.0131	Unknown
Ethylenediamine	+1.44	0.0175	Amino acid metabolism (histamine H1 receptor)
Propanoic acid	+1.41	0.0201	Krebs cycle; amino acid metabolism (phenylalanine metabolism)
Dodecanoic acid	+1.30	0.0331	Fatty acid biosynthesis
d-Gluconic acid	−1.63	0.0056	Glucose metabolism Inositol phosphate metabolism
Glucose oxime hexakis	−1.40	0.0201	Glucose metabolism
Propanedioic acid	−1.40	0.0201	Glucose metabolism; fatty acid synthesis
(R*,S*)-3,4-Dihydroxybutanoic acid	−1.37	0.023	Unknown
d-Mannitol	−1.37	0.023	Glucose metabolism
3-(3-Hydroxyphenyl)-3-hydroxypropionic acid	−1.34	0.0261	Glucose metabolism
Glycine	−1.32	0.0163	Glutathione metabolism; amino acid metabolism
Allonic acid	−1.29	0.0331	Glucose metabolism
Pentaric acid	−1.27	0.037	Krebs cycle
Benzeneacetic acid	−1.25	0.0412	Amino acid metabolism (phenylalanine metabolism)
Lyxose	−1.22	0.0456	Glucose metabolism
^ 1^H-Indole-3-acetic acid	−1.13	0.0656	Amino acid metabolism (tryptophan metabolism)

*Note*. “+” and “−” indicate that compared with the control group, the relative concentrations of metabolites were, respectively, increased and decreased in the presbycusis group.
